# Role of Radiotherapy in an Inflammatory Pseudotumor of the Maxillary Sinus and Imaging Findings: A Case Report

**DOI:** 10.7759/cureus.109128

**Published:** 2026-05-18

**Authors:** Ricardo Mendoza Coronado, Ana Karen Luna Marroquin, Laura Estefany Purata Hernandez, Rafael Piñeiro Retif, Oscar Vidal Gutierrez

**Affiliations:** 1 Radiation Oncology, Hospital Universitario “Dr. José Eleuterio González”, Universidad Autónoma de Nuevo León, Monterrey, MEX; 2 Radiology and Imaging, Hospital Universitario “Dr. José Eleuterio González”, Universidad Autónoma de Nuevo León, Monterrey, MEX; 3 Anesthesiology, Hospital Universitario “Dr. José Eleuterio González”, Universidad Autónoma de Nuevo León, Monterrey, MEX; 4 Oncology, Hospital Universitario “Dr. José Eleuterio González”, Universidad Autónoma de Nuevo León, Monterrey, MEX

**Keywords:** case report, head and neck radiology, magnetic resonance imaging, maxillary sinus pseudotumor, paranasal sinus lesion, radiology, radiotherapy

## Abstract

Inflammatory pseudotumor (IPT) is a benign lesion of inflammatory origin that can mimic a malignant neoplasm because of its aggressive clinical and imaging behavior, particularly in uncommon locations such as the paranasal sinuses.

In this article, we present the case of a patient diagnosed with IPT of the left maxillary sinus, which demonstrated extension into adjacent soft tissues and secondary involvement of cranial nerves due to extensive local invasion. Findings on computed tomography (CT) and magnetic resonance imaging (MRI) revealed bone destruction and extension into neighboring anatomical structures, including the orbital floor, masticatory space, and temporomandibular joint, without imaging evidence of perineural spread. These imaging findings were essential in delineating the lesion and guiding the therapeutic approach.

## Introduction

Inflammatory pseudotumor (IPT) is a benign mass that, as its name indicates, is of inflammatory origin; however, one of the reasons it is referred to as a “pseudotumor” is that, clinically and radiologically, it presents in an invasive manner and therefore is often confused with a malignant tumor [1]. Its etiology is usually uncertain, and histologically, it is characterized by a large number of plasma cells, fibrous cells, lymphocytes, eosinophils, and histiocytes [2].

It may be observed in any part of the body; however, it most commonly involves the lung. Other structures that may be affected by this condition include the liver, mesentery, spleen, and intestine [3].

In the literature, these lesions have also been described using various terms, including inflammatory myofibroblastic tumor, plasma cell granuloma, histiocytoma complex, and fibrous xanthoma [2]. However, it is important to note that the term IPT is used broadly to describe inflammatory lesions with a tumor-like appearance, whereas inflammatory myofibroblastic tumor is currently recognized as a distinct neoplastic entity; therefore, the two terms should not be considered strictly interchangeable.

Some of the challenges posed by this condition are that, when it involves the nasal cavity and paranasal sinuses, it is often associated with a high rate of recurrence, has considerable destructive potential, and its diagnosis is frequently overlooked or incorrect because of the lack of specific signs. In addition, few cases have been documented, most of which date back many years, and there is no clear consensus on the optimal treatment for this pathology. In particular, involvement of the maxillary sinus has been rarely described in the literature. This case adds value by documenting the role of radiotherapy and detailing the imaging findings - areas that remain limited in previous reports - thereby contributing to the expansion of available evidence and potentially guiding future therapeutic decisions.

## Case presentation

This case involves a 40-year-old female patient with a history of long-standing hypertension. She has no relevant family medical history and no personal history of sinusitis, autoimmune diseases, immunosuppression, trauma, or previous surgical procedures. In November 2022, she began experiencing profuse epistaxis, for which she consulted multiple primary care physicians who diagnosed her with “nasal dryness” and prescribed only symptomatic treatment. This consisted of initial management measures for epistaxis, including nasal compression, anterior head positioning, and application of local cold, as well as the occasional use of topical vasoconstrictors and anterior nasal packing in persistent episodes. Additionally, nasal saline irrigations were indicated for the management of nasal dryness. These interventions were performed intermittently from November 2022 to July 2023; however, precise information regarding dosages, treatment duration, and specific medications is not available because this information could not be accurately recalled by the patient.

Due to the lack of improvement despite adherence to treatment, and the subsequent development of predominantly left-sided nasal congestion associated with diplopia, blurred vision, and paresthesias of the left cheek and upper lip, she decided to seek evaluation by an otorhinolaryngologist in July 2023. On physical examination, the patient was found to have an irregular, friable, pink-colored mass in the left nasal cavity, associated with edema and congestion of the adjacent mucosa; therefore, laboratory tests and imaging studies were requested.

On ophthalmologic examination, the patient presented with binocular diplopia, blurred vision, and left-sided ophthalmoplegia, with the left eye resting in abduction (exotropia). Ipsilateral ptosis was observed, consistent with involvement of the oculomotor nerve. The pattern of extraocular movement impairment suggested additional compromise of the trochlear and abducens nerves. The patient also reported left-sided facial pain with neuropathic characteristics, along with paresthesias over the left cheek and upper lip, consistent with involvement of the trigeminal nerve.

A non-contrast computed tomography (CT) scan of the paranasal sinuses, performed on August 1, 2023, documented a poorly defined hypodense lesion at the level of the left maxillary sinus, measuring 66 × 40 × 52 mm and showing bone destruction of the anteromaxillary walls, as well as the ipsilateral nasal cavity, with apparent involvement of the orbital muscles. However, given that this was a non-contrast study and considering the aggressive characteristics of the lesion, a contrast-enhanced magnetic resonance imaging (MRI) study of the paranasal sinuses was recommended for further evaluation and assessment of its extent (Figures 1-2).

**Figure 1:**
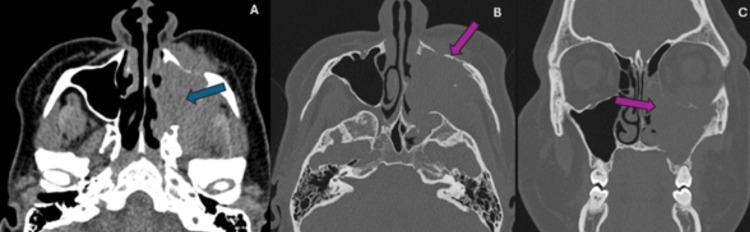
A) Axial section with soft tissue window, B) axial section with bone window, C) coronal reconstruction with bone window. A poorly defined hypodense lesion is identified at the level of the left maxillary sinus (blue arrow), initially drawing attention because of its destructive osseous behavior involving the maxillary antrum and the ipsilateral nasal cavity (pink arrow).

**Figure 2:**
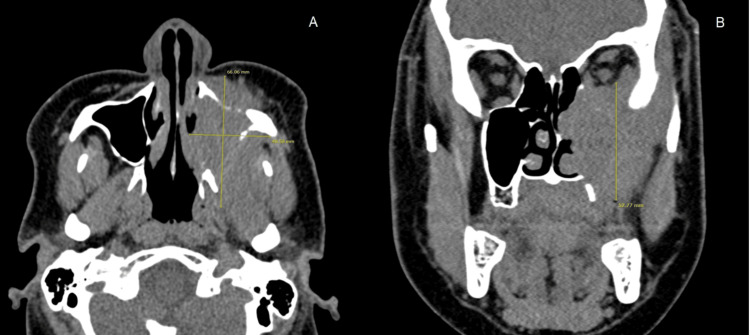
A) Axial non-contrast CT of the paranasal sinuses, B) coronal reconstruction. A poorly defined hypodense lesion is identified, centered in the left maxillary sinus on axial images (A), measuring approximately 66 × 40 mm in the anteroposterior and laterolateral dimensions. Coronal reconstruction (B) demonstrates the craniocaudal extent of the lesion, with an approximate length of 52 mm. CT: computed tomography

A contrast-enhanced MRI study of the paranasal sinuses was performed, which revealed an irregular, poorly defined lesion in the left maxillary sinus, hypointense on T1-weighted images, heterogeneous on T2-weighted images, and showing heterogeneous contrast enhancement, with local extension to adjacent structures. Involvement of the maxillary sinus walls, ipsilateral nasal cavity, orbital floor, and inferior extraocular musculature was observed, as well as extension into the masticatory space, alveolar processes, and the temporomandibular joint. These findings suggested, as the primary diagnostic consideration, invasive granulomatous fungal sinusitis of the left maxillary sinus with associated soft tissue involvement. The apparent involvement of cranial nerves III, IV, V, and VI was interpreted as secondary to the extensive involvement of adjacent anatomical structures, without imaging evidence of perineural spread. However, sinonasal squamous cell carcinoma or adenoid cystic carcinoma could not be excluded (Figure 3).

**Figure 3:**
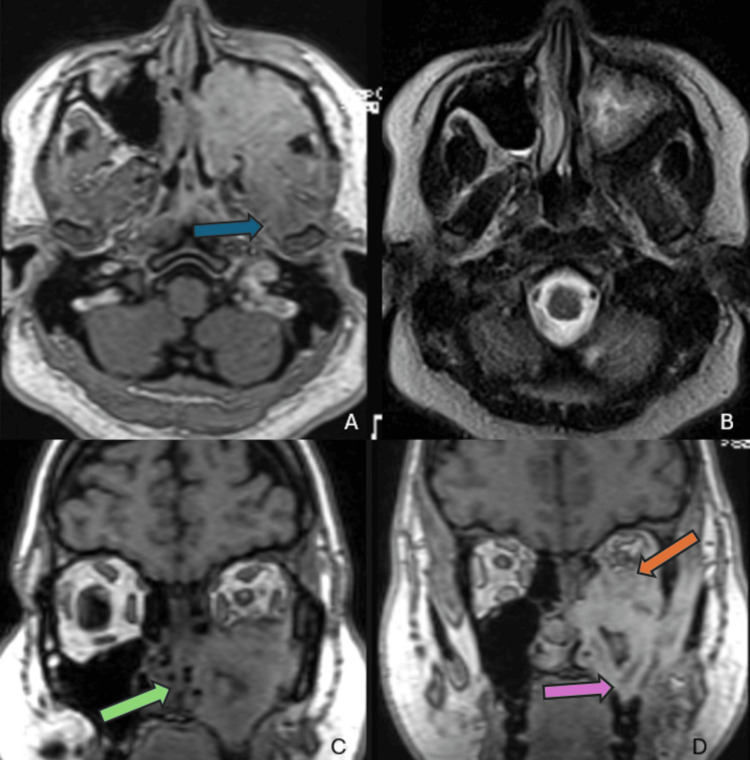
A) Contrast-enhanced axial T1, B) non-contrast axial T2, C) non-contrast coronal T1, D) contrast-enhanced coronal T1. An irregular, poorly defined lesion is observed in the left maxillary sinus, hypointense on T1-weighted images (C), heterogeneous on the T2 sequence (B), with heterogeneous contrast enhancement (A and D), involving the walls of the maxillary antrum, the ipsilateral nasal cavity (green arrow), the floor of the orbit (orange arrow), the inferior extraocular muscle complex, the alveolar processes (purple arrow), the masticator space, and the temporomandibular joint (blue arrow).

Two weeks later, on September 9, 2023, the patient underwent surgical resection of the lesion; however, complete excision could not be achieved. Intraoperative biopsy revealed an acute and chronic inflammatory process with predominance of plasma cells. Histochemical stains, including periodic acid-Schiff (PAS), Grocott-Gomori methenamine silver, Giemsa, and Warthin-Starry, were all negative for microorganisms. Immunohistochemical analysis demonstrated CD3 expression with a reactive pattern, negativity for TIA1, and negativity for ALK. Immunostaining for IgG and IgG4 showed a preserved ratio without a significant increase in IgG4-positive cells. Smooth muscle actin was positive in myofibroblasts. Based on these findings, infectious processes, IgG4-related disease, lymphoma, and ALK-positive inflammatory myofibroblastic tumor were excluded. Overall, the findings were consistent with an IPT.

Following the surgical procedure performed on September 9, 2023, the patient was referred to the Department of Otorhinolaryngology, where she was placed under close surveillance and initiated on high-dose corticosteroid therapy with oral prednisone at 100 mg daily (50 mg twice daily) for eight weeks. This approach was chosen due to incomplete tumor resection and persistence of symptoms, including epistaxis, diplopia, and blurred vision.

Because complete resection of the lesion was not achieved during the initial procedure and the patient remained symptomatic, she underwent a second surgical intervention on January 18, 2024. However, due to tumor extension and infiltration, only partial tumorectomy could again be performed. Repeat biopsy of the lesion was obtained, and histopathological examination of the surgical specimen from the maxillary sinus and inferior turbinate revealed findings consistent with IPT. Immunohistochemistry for smooth muscle actin was positive in myofibroblasts. Residual foci of the lesion, with features similar to those observed in the previous biopsy, were identified, without evidence of neoplasia. Therefore, neoplastic processes were excluded, and the diagnosis of IPT was confirmed.

In February 2024, contrast-enhanced CT of the paranasal sinuses demonstrated a residual lesion in the left maxillary sinus measuring 4.2 × 3.4 × 3.7 cm (Figure 4).

**Figure 4:**
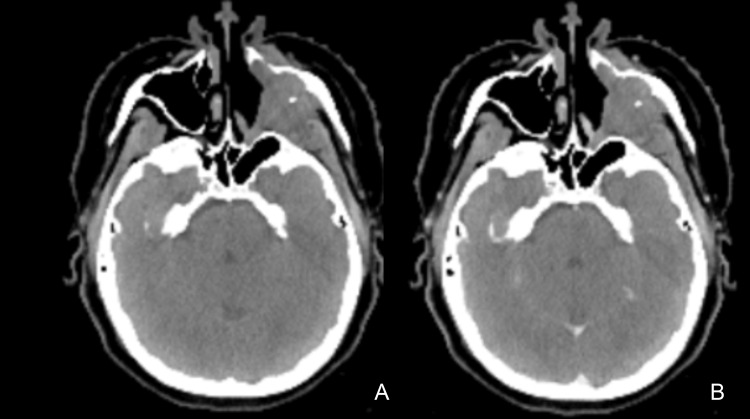
A) Non-contrast axial CT scan of the paranasal sinuses, B) contrast-enhanced axial CT scan of the paranasal sinuses. Computed tomography (CT) demonstrating persistence of a previously diagnosed inflammatory pseudotumor in the left maxillary sinus, appearing as a poorly defined hypodense lesion with mild post-contrast enhancement.

However, due to failure of the initial surgical management and the absence of symptomatic response to high-dose corticosteroids, along with persistent disease activity evidenced on postoperative imaging studies, the patient was referred to the Radiation Oncology Department for treatment of residual disease.

Definitive radiotherapy was delivered to the residual lesion with a total dose of 30 Gy in 15 fractions (2 Gy per fraction). This dose was selected given the lack of standardized treatment protocols in the literature, where reported doses range widely from 8 Gy to 60 Gy, and no specific radiotherapy technique has been established. A conservative dosing strategy was therefore adopted to ensure adequate tolerance of organs at risk, particularly considering the potential need for future reirradiation.

Treatment was delivered using a Varian Medical Systems Halcyon linear accelerator with volumetric modulated arc therapy (VMAT). The gross tumor volume (GTV) was defined based on contrast-enhanced T1-weighted MRI and contrast-enhanced CT of the head and neck. The clinical target volume (CTV) was considered equivalent to the GTV, given the non-metastatic behavior of the disease, with a measured volume of 47.9 cm³. A 5-mm margin was added to account for setup uncertainty, generating a planning target volume (PTV) of 113.0 cm³. For treatment simulation, contrast-enhanced CT of the head and neck was performed using a five-point thermoplastic immobilization mask. Daily image guidance was carried out using cone-beam CT (CBCT) to verify accurate patient positioning and target localization. Critical organs at risk were carefully spared, particularly the optic pathways, spinal cord, and brainstem. The maximum recorded doses (Dmax) were 30.78 Gy for the optic structures, 2.25 Gy for the spinal cord, and 12.93 Gy for the brainstem. Radiotherapy was successfully completed in May 2024.

In July 2024, two months after completion of radiotherapy, the patient attended a follow-up visit and reported pruritus within the irradiated field without visual disturbances, as well as xerostomia without dysphagia. In October 2024, she reported persistent vertical diplopia only during binocular vision, which was not present during monocular vision. She also experienced self-limited headaches that did not require treatment, with resolution of the remaining symptoms.

At the follow-up visit in January 2025, the patient reported symptoms similar to those previously described. Contrast-enhanced MRI of the paranasal sinuses performed in the same month demonstrated structural changes in the left maxillary sinus secondary to prior antrostomy, with evidence of reparative fibrotic changes involving the lateral walls and orbital floor without other significant findings (Figure 5).

**Figure 5:**
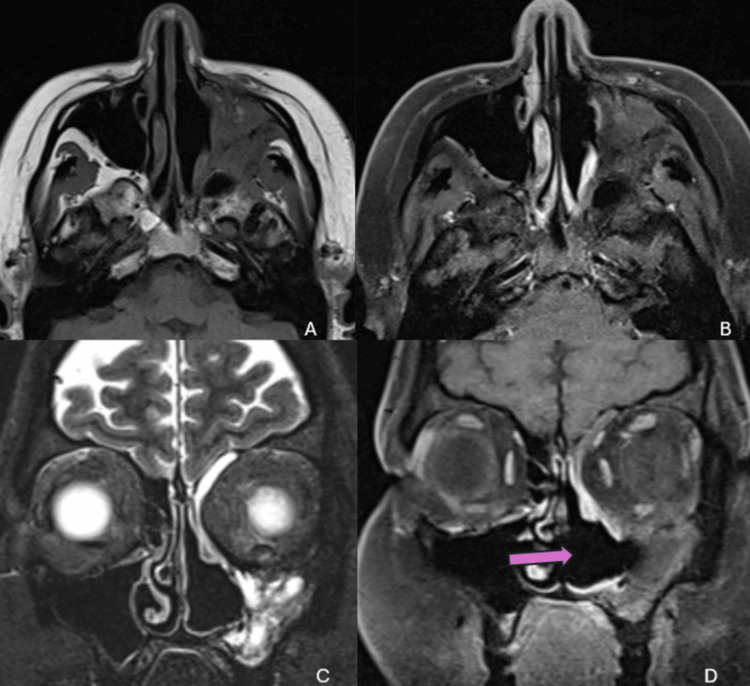
A) Non-contrast axial T1, B) contrast-enhanced axial T1, C) coronal T2, D) contrast-enhanced coronal T1. Post-treatment changes in the left maxillary sinus were noted, characterized by the absence of the middle and inferior turbinates (pink arrow), with irregular tissue occupying the maxillary sinus. This tissue demonstrated hypointensity on T1-weighted images (A), hyperintensity on T2-weighted images (C), and, importantly, showed no post-contrast enhancement (B and D). There was no associated mass effect, bone destruction, or progression compared to prior imaging. These imaging characteristics, particularly the lack of enhancement and absence of aggressive features, favored post-treatment fibrotic changes rather than residual active disease or viable tumor.

At the most recent follow-up in November 2025, the patient reported persistent vertical diplopia exclusively during binocular vision, absent during monocular viewing. MRI performed during the same visit showed fibrotic changes without evidence of residual or recurrent disease.

## Discussion

In this case report, we included the treatment received by a patient diagnosed with IPT with plasma cell predominance of the left maxillary sinus at our tertiary care center, as well as its imaging characteristics, visualized by CT and MRI.

Cases of IPT have been reported in almost all organs and sites of the body; however, the lung is the most commonly affected site and was the first location in which it was described in 1940 [4]. When it occurs in the head and neck region, it usually accounts for less than 5% of cases [2]; nevertheless, it is important to consider it. The main symptoms presented by patients with this condition are nasal congestion, facial discomfort, epistaxis, and ocular symptoms [5]. On physical examination, a nonspecific sinonasal mass is usually found, which progressively worsens over months or years. On endoscopy, a mass covered by normal mucosa, a polyp in the middle meatus, or hemorrhagic rhinorrhea may be visualized [6].

Radiologically, it shows very nonspecific findings; on non-contrast CT, a predominantly heterogeneous lesion is usually identified; in a minority of cases, it may be homogeneous, and calcifications within the lesion are rarely found. It may present with bone destruction or invasion of the nasal cavity, infratemporal fossa, sphenoidal region, maxillofacial region, ethmoidal sinuses, or even intracranially. On MRI, T1-weighted images show lesions that are isointense to muscle, and on T2-weighted sequences, the lesions are usually mixed isointense to mildly hyperintense relative to muscle. After contrast administration, mild to moderate enhancement may be observed [7]. In contrast to this general pattern described in the literature, the present case demonstrates a markedly aggressive radiological behavior, characterized by a poorly defined lesion in the left maxillary sinus measuring 66 × 40 × 52 mm, with extensive bony destruction of the anterolateral maxillary walls and involvement of the ipsilateral nasal cavity. Furthermore, there is extension into critical adjacent structures, including the orbital floor and inferior extraocular muscles, as well as invasion of the masticatory space, alveolar processes, and temporomandibular joint. On MRI, the lesion demonstrates heterogeneous T2 signal, T1 hypointensity, and heterogeneous post-contrast enhancement; however, the extent of local infiltration is significantly greater than typically described for IPT, suggesting a more aggressive biological behavior and necessitating consideration of alternative diagnoses such as invasive fungal disease or malignant neoplasms. Differential diagnoses to be considered include carcinoma, aggressive fibrous lesions, lymphoma, and chronic fungal disease.

As previously mentioned, there is no way to confirm the diagnosis through imaging studies or physical examination alone; therefore, intraoperative biopsy plays a fundamental role in this condition.

Treatment options include surgery, chemotherapy, or radiotherapy, in addition to biopsy or resection as initial treatment [8], which may be used alone or in combination. The treatment of choice in this presentation is surgery; corticosteroids are considered second-line when complete excision is not possible. Radiotherapy is mainly used in patients in whom surgery or corticosteroid therapy has not been successful or is contraindicated [9], and it has even been found that treatment with this modality is more successful in cases of resistant, refractory, or recurrent pseudotumor [10]. Conventional fractionated radiotherapy has historically been delivered to doses in the range of 40-50 Gy in 1.8-2 Gy fractions, which has been considered an effective therapeutic approach in selected reports [11]. However, it is important to emphasize that there is no universally standardized radiotherapy regimen for this entity, and the available evidence is heterogeneous, with reported dose schedules ranging widely from 8 Gy to 60 Gy depending on tumor characteristics, anatomical location, and proximity to critical structures. In this context, the selection of a lower total dose in the present case must be interpreted as an individualized, risk-adapted modification of standard practice rather than a deviation from evidence-based principles. Dose de-escalation was driven by the close proximity of critical organs at risk, including the optic apparatus, brainstem, and spinal cord, as well as the relatively large PTV and the need to preserve the possibility of future reirradiation.

## Conclusions

IPT of the maxillary sinus, although infrequent, should be considered in the differential diagnosis of sinonasal masses with aggressive features on imaging studies. CT and MRI are essential not only for delineating the extent of the lesion but also for guiding possible differential diagnoses and planning therapeutic strategies. In the case described, imaging allowed identification of findings suggestive of perineural invasion and bone destruction, which supported a multidisciplinary management approach. Radiotherapy played an important role in local disease control, particularly in the setting of incomplete surgical resection and partial response to corticosteroid therapy. In this context, the lesion can be considered clinically refractory, as it demonstrated persistent disease activity despite multimodal initial management, including surgical intervention and systemic corticosteroid treatment, both of which failed to achieve complete disease resolution and required subsequent therapeutic escalation.

The administration of 30 Gy in 15 fractions using VMAT was well tolerated and was associated with subsequent clinical and radiological improvement, with imaging findings demonstrating fibrotic changes suggestive of a favorable treatment response. However, given the multimodal nature of management in this case - including surgery, corticosteroid therapy, and radiotherapy - the observed outcome likely reflects a combined therapeutic effect rather than the isolated impact of any single modality. This case supports the role of radiotherapy as part of a multidisciplinary treatment strategy for clinically refractory IPT of the maxillary sinus and highlights the importance of radiological follow-up for short- and intermediate-term disease monitoring.
